# Research hotspots and trends of the tele-rehabilitation for stroke survivors based on CiteSpace: A review

**DOI:** 10.1097/MD.0000000000033398

**Published:** 2023-03-31

**Authors:** Linzhang Li, Yan Sun

**Affiliations:** a Geriatric Medical Center, Ward 3, Wenjiang People’s Hospital of Chengdu, Chengdu, China; b School of Medicine, University of Electronic Science and Technology of China, Chengdu, China; c Sichuan Academy of Medical Sciences & Sichuan Provincial People’s Hospital, Chengdu, China.

**Keywords:** bibliometrics, CiteSpace, remote rehabilitation, stroke, visual analysis

## Abstract

Our first goal is to understand the research status and popularity of telerehabilitation research for stroke survivors since 2012; the second goal is to analyze the research trends and frontiers in this field, and provide a scientific basis for the future application of telerehabilitation technology in patients with poststroke functional defects. We searched the Web of Science Core Collection (WoSCC) for literature on telerehabilitation for stroke survivors published from 2012 to 2022. The included articles were visually analyzed using CiteSpace6.1.6R (64-bit). In total, 968 eligible articles were included in this study. In the past 10 years, the number of papers published on telerehabilitation after stroke has been increasing annually, with the largest number of papers published in the United States and Australia, with 101 papers published by Chinese scholars. Some subsets of cooperative networks have been formed among major research institutions and their authors, but the scale remains small, and academic exchanges and cooperation need to be strengthened further. Research on virtual reality (VR) technology and rehabilitation robot technology is popular, and the choice of time and intensity of rehabilitation exercises, patients’ participation in rehabilitation exercises, and care are also worth attention. In the last 10 years, research on telerehabilitation technology in the field of rehabilitation for stroke survivors has steadily developed, and is characterized by multidisciplinary joint development. Countries around the world can combine their own characteristics and advantages, strengthen academic exchanges and cooperation with mature research institutions or authors, and explore suitable poststroke remote rehabilitation technologies and service models in different environments.

## 1. Introduction

According to the World Stroke Organization, the total number of chronic stroke survivors worldwide is estimated at 80 million, and over 13.5 million people experience stroke each year, approximately 40% of whom require recovery.^[1]^ In 2019, approximately 17.04 million people aged 40 and above had or once had experienced a stroke in China. In 2018, 1.94 million people died of stroke. Stroke has become the leading cause of death and disability among adults in China.^[2]^ The incidence of stroke may continue to increase due to the aging of the population.^[3]^ Approximately 75% of survivors have functional impairments in the domains of language, movement, and perception,^[4]^ putting great pressure on society and the family.^[5,6]^ Considering factors such as economy, transportation distance, disease, and long-term care, many stroke patients return to the community or family to continue to recover in the acute phase until their illness is stable.^[7,8]^ Continuous rehabilitation after stroke has been confirmed to effectively promote the recovery of motor function and the improvement of daily living ability in patients.^[9,10]^ In China, the community health care service capacity is insufficient because of a lack of community rehabilitation resources and talent,^[11]^ and the rehabilitation needs of stroke survivors in home or community backgrounds cannot be met.

In recent years, owing to the exploration of remote rehabilitation technology and the popularity of remote rehabilitation programs and models, remote rehabilitation with the help of computer technology, information and communication technology, mobile network technology, and rehabilitation therapy can overcome spatial barriers, expand the scope of rehabilitation resources, and enhance doctor-patient communication with stroke survivors in families, communities, and remote areas. It can also avoid transportation inconveniences for patients and provide professional rehabilitation guidance and advice.^[12–14]^ Telerehabilitation is commonly defined as the use of electronic information and communication technologies to provide and support health care when distance separates the participants, covering a range of healthcare services such as assessment, testing, intervention, supervision, education and counseling.^[15]^ There are different forms of telerehabilitation, which can be synchronous, asynchronous, or a combination of both. Synchronous telerehabilitation is a communication technology based on real-time data transmission in both directions, allowing the patient and therapist to be online simultaneously so that the therapist can provide real-time rehabilitation services to the patient. Asynchronous telerehabilitation is based on “storage” technology, where the therapist stores information in text or video form on a “server” and the patient performs self-exercise according to the instructions.^[16,17]^ Functional recovery and improvement in stroke survivors is usually over a long time window, so individual rehabilitation needs, treatment goals and plans must be redefined periodically.^[18]^ The future of telerehabilitation faces both opportunities and obstacles and challenges. The clinician or physical therapist does not have face-to-face access to the patient, which can lead to a lack of quantitative and objective assessment of the patient.^[19]^ The trait of technology dependence may hinder the development of telerehabilitation to some extent.^[20]^ The accessibility of technology for elderly patients, low education level groups, and low income groups may affect their participation and motivation.^[15,21]^ On the other hand, the COVID-19 pandemic has given a boost to telemedicine, which can be the complementary service of choice in the postCOVID-19 era.^[22]^ Policy assurance from the health sector is essential. Today, many countries have developed guidelines and information for conducting telemedicine, which is undoubtedly beneficial for telehealth care.^[15]^ In addition, although some therapists have suggested that data security, privacy, and confidentiality may also affect the spread of telehealth,^[23]^ 1 study confirmed the satisfaction and acceptance of telehealth among patients, who claimed that personal privacy was protected.^[24]^

To gain a comprehensive understanding of the current status and hot spots of poststroke remote rehabilitation research in the past decade and to explore the frontiers of future development, this study visually analyzed the literature on stroke telerehabilitation in the Web of Science database through CiteSpace, hoping to provide a basis for subsequent in-depth research in this field in the future.

## 2. Methods

### 2.1. Ethic statement

Ethical approval is not required for this study because it is conducted based on secondary data.

### 2.2. Data source

**Figure 1.**
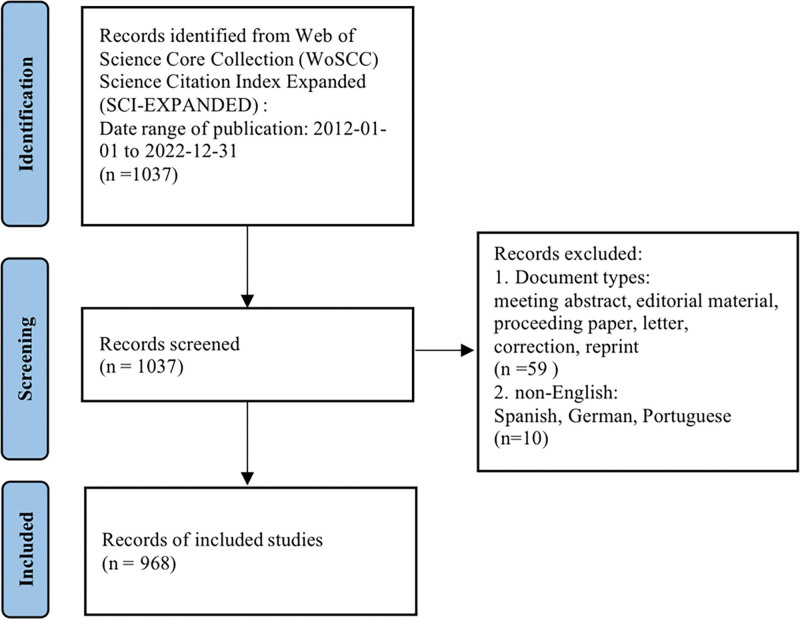
Study data retrieval flow chat. The database searched was Web of Science Core Collection, searched from January 1, 2012 to December 31, 2022. 59 records such as meeting abstracts, editorial materials, proceeding papers, letters, corrections, and reprints were excluded; 10 nonEnglish records were excluded in total. After title and abstract review, 968 records that met the study criteria were finally included.

With the Web of Science Core Collection (WoSCC) Science Citation Index Expanded as the retrieval source, the following topics were searched: “stroke * OR apoplex * OR (cerebrovascular accident) OR (brain vascular accident) OR (cerebral infarction) OR (cerebral hemorrhage) OR (subarachnoid hemorrhage)” AND topic: “telerehabilitation OR tele-rehabilitation OR (remote rehabilitation) OR (online rehabilitation) OR (web-based rehabilitation).” Articles published in English from January 1, 2012, to December 31, 2022, were retrieved. The literature types included original articles, review articles, early access, and book chapters, excluding meeting abstracts, editorial materials, proceedings papers, correspondences, corrections, and reprints. In total, 968 articles were included. Figure 1 illustrates the flow of the literature search.

### 2.3. Research method

Visual analysis software CiteSpace 6.1. R 6 (64-bit) was used as a research tool. CiteSpace, written in the Java language, draws a visual knowledge map based on the co-citation analysis theory and pathfinding network algorithm to present potential knowledge, rules, and distribution contained in the scientific literature. It helps researchers to explore the knowledge base, research hotspot and frontiers of a certain research field.^[25]^ Previous studies have proved that CiteSpace has become a relatively influential analysis software in the field of bibliometrics.^[26–28]^ The WoSCC has been included in the literature selection record content as “Full record and cited reference” in plain text file format for export. The downloaded files were renamed as download 1. txt and download 2. txt. Running CiteSpace, importing text files, removing duplicates, and transcoding yielded 0 duplicate documents and 968 valid documents. The project (Project) was created, and the parameters were set. The time section (Time Slicing) was defined from 2012 to 2022, with every 1 year as 1 time slice; Node types included the following: Author, Country, Institution, Keyword, and Reference. These nodes yielded an author cooperative network knowledge map, a national cooperative network map, an institution cooperation network knowledge map, a keyword co-occurrence knowledge map, and a literature co-cited knowledge map. The selection criteria were defined as follows: top N = 50; pruning was conducted as follows: tracing was selected (Pathfinder), the section network was switched (Pruning sliced networks), and the merged network was pruned to obtain a clear prominent network map.^[29]^

## 3. Results

### 3.1. Basic statistical analysis

**Figure 2.**
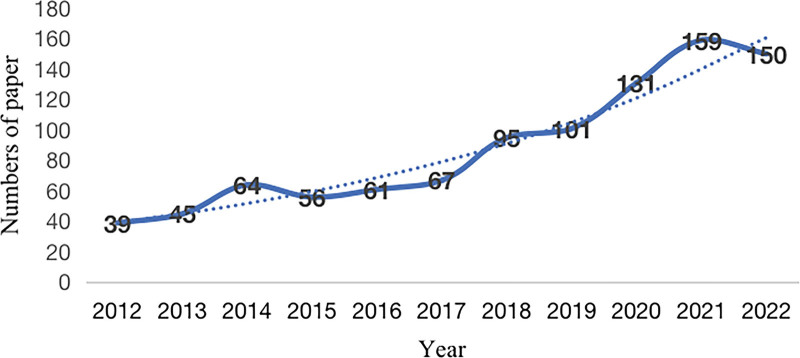
Publication trends over time (2012–2022). The horizontal coordinate is the year of publication and the vertical coordinate is the number of papers. The blue curve represents the change in the number of publications, and the blue dashed curve reflects the trend of the change.

The analysis of the basic information of the 968 included documents showed that the number of publications in the field of telerehabilitation after stroke has been increasing yearly in the past 10 years (Fig. 2). The number of articles increased relatively slowly from 2012 to 2017. In 2018, the number of articles grew rapidly, and by 2021, it had reached its peak, with 159 articles published.

### 3.2. Journal distribution

The top 5 journals from 2012 to 2022 are the *Journal of Neuroengineering and Rehabilitation* (36), accounting for 3.719% of papers; *Disability and Rehabilitation* (33), accounting for 3.409% of papers; *Frontiers in Neurology* (33), accounting for 3.409% of papers; *Sensors* (27), accounting for 2.789% of papers; and *Archives of Physical Medicine and Rehabilitation* (23), accounting for 2.376% of papers. Table 1 shows the details of other major distribution magazines. The study of telerehabilitation in stroke survivors intersects with the following nonmedical fields: engineering, electrical and electronic, sport science, computer science, information system, and telecommunication.

**Table 1:** Top 10 journals by publications.

Rank	Journal title	Amount	Country	IF	Research area
1	*Journal of Neuroengineering and Rehabilitation*	36	England	5.208	Neurosciences (Q2) Rehabilitation (Q1)
2	*Disability and Rehabilitation*	33	England	2.439	Rehabilitation (Q2)
3	*Frontiers in Neurology*	33	Switzerland	4.086	Clinical Neurology (Q2)
4	*Sensors*	27	Switzerland	3.847	Engineering, Electrical & Electronic (Q2)
5	*Archives of Physical Medicine and Rehabilitation*	23	USA	4.06	Sport Science (Q1) Rehabilitation (Q1)
6	*BMJ Open*	22	England	3.007	Medicine, General & Internal (Q2)
7	*PLoS One*	22	USA	3.752	Multidisciplinary Sciences (Q2)
8	*Topics in Stroke Rehabilitation*	20	England	2.177	Rehabilitation (Q3)
9	*Aphasiology*	18	England	1.902	Clinical Neurology (Q4) Rehabilitation (Q3)
10	*IEEE Access*	15	USA	3.476	Computer Science, Information System (Q2) Engineering, Electrical & Electronic (Q2) Telecommunication (Q2)

IF = impact factor.

### 3.3. Country distribution

**Figure 3.**
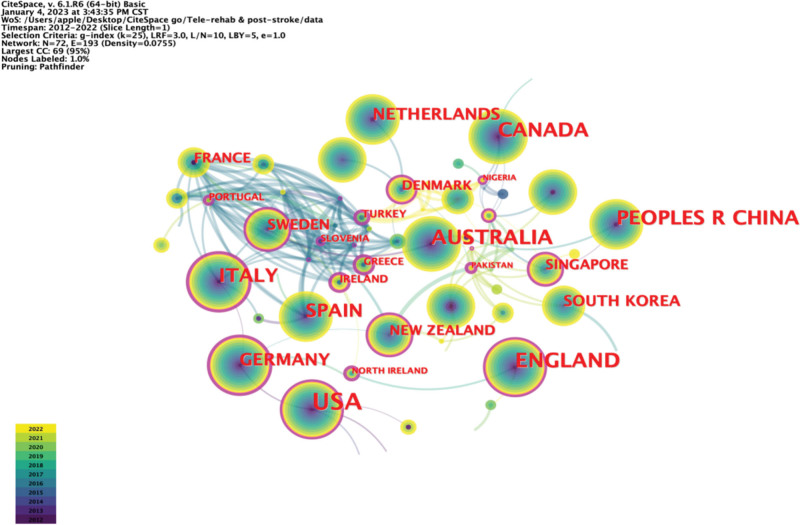
National collaborative network mapping. A node represents a country, and the size of the node indicates the number of publications in that country. The color of each layer of the node represents the year of publication, and the outermost purple color indicates that the centrality of the country is >0.1. The lines between the nodes represent the cooperation between countries, and the color and thickness of the lines indicate when the cooperation started and how close the cooperation is, respectively.

Related studies on the application of telerehabilitation technology in the rehabilitation treatment of patients after stroke are concentrated in Europe, the United States, and Oceania. In the past 10 years, the United States has published the most articles, totaling 259 articles; 118 articles were published in Australia and 111 articles were published in England. The top ten countries and regions in this field are the United States, Australia, England, China, Canada, Italy, Spain, Germany, the Netherlands, and Switzerland (Table 2). Figure 3 depicts the cooperative network relationships between publishing countries. A circle represents a country, and the size of the circle directly correlates with the number of articles. The connection represents cooperation between countries. The outer purple circle indicates the country high centrality and its prominent role in the collaborative network. As shown in Figure 3, the connections between other European countries such as Italy, Sweden, Portugal, and Greece are much tighter than those between other regions.

**Table 2:** Top 10 countries by publications.

Rank	Country	Number of publications
1	USA	259
2	Australia	118
3	England	111
4	China	101
5	Canada	97
6	Italy	88
7	Spain	67
8	Germany	49
9	Netherlands	43
10	Switzerland	35

### 3.4. Institutional distribution

**Figure 4.**
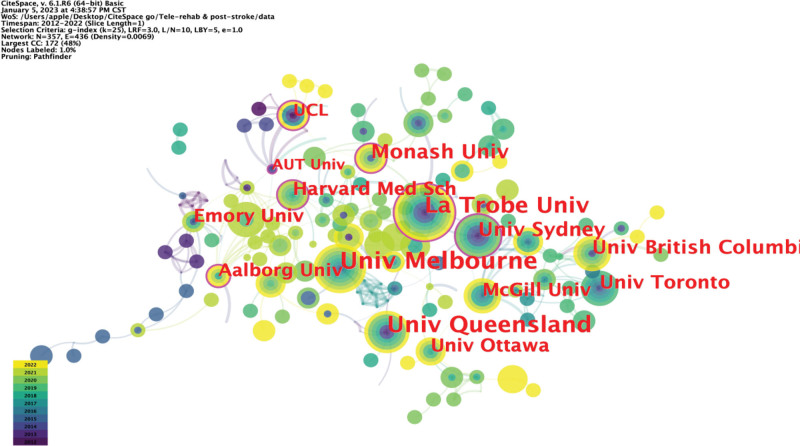
Institution collaboration network mapping. A node represents an institution, and the size of the node indicates the number of publications of this institution. The color of the node represents the year of the publication, and the outer purple color indicates that the centrality of the institution is >0.1. The lines between the nodes represent the cooperation between institutions, and the color and thickness of the lines indicate when the cooperation started and how close the cooperation is.

The number of research institutions shows that European and Oceanian countries have conducted in-depth research and exploration in this field. The top ten organizations with the most publications are the University of Melbourne, University of Queensland, La Trobe University, Monash University, University of Sydney, The University of British Columbia, University of Toronto, University of Ottawa, McGill University, and Harvard Medical School (Table 3). These institutions have long been engaged in research on telerehabilitation technology in stroke rehabilitation treatment and have strong academic abilities and international influence. The main research institution knowledge map shows 357 nodes with 436 connections at a density of 0.069 (Fig. 4). Each circle represents a research institution. The size of the circle is positively correlated with the number of articles in the institution. The connection between the circles represents cooperation between the institutions, the color of the connection represents the beginning of cooperation between the institutions, and the thickness of the connection represents the strength of the collaboration. The purple outline of the periphery of the circle indicates that the centrality of the mechanism exceeds 0.1. Nodes with centrality exceeding 0.1 are called key nodes and are significant in the structure of network relationships. Visual analysis identified 7 institutions with centrality >0.1: University College London, Monash University, Aalbory University, AUT University, La Trobe University, University of Sydney, and Harvard Medical School.

**Table 3:** Top 10 institutions by publications.

Rank	Institution	Country	Amount	Institution	Country	Centrality
1	University of Melbourne	Australia	26	UCL	England	0.18
2	University of Queensland	Australia	23	Monash University	Australia	0.17
3	La Trobe University	Australia	21	Aalbory University	Denmark	0.17
4	Monash University	Australia	15	AUT University	New Zealand	0.17
5	University of Sydney	Australia	14	La Trobe University	Australia	0.16
6	University of British Columbia	England	13	University of Sydney	Australia	0.12
7	University of Toronto	Canada	12	Harvard Medical School	USA	0.12
8	University of Ottawa	Canada	11	Emory University	USA	0.09
9	McGill University	England	11	University of Melbourne	Australia	0.07
10	Harvard Medical School	USA	11	University of Ottawa	Canada	0.06

### 3.5. Author collaborative analysis

**Figure 5.**
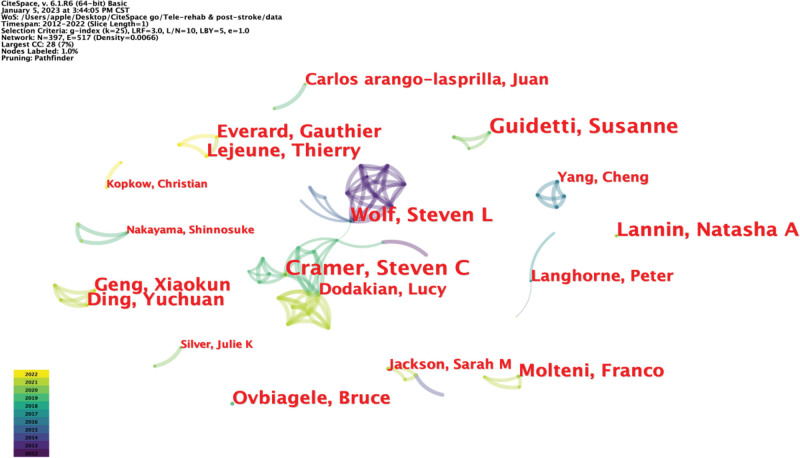
Author collaboration network mapping. The connecting line represents the collaboration between the 2 authors, and the color of the connecting line indicates when the collaboration first began. The network line formed by the connecting lines is the set of collaborations formed between the authors.

We identified the core authors according to Price Law.^[30]^ The minimum number of published papers is M, and M ≈ 0.749 (Nmax 1/2). Nmax is the total number of papers published by the most published authors in a particular research field. The CiteSpace analysis shows that Guidetti S and Gramer Steven C are the authors with the most publications (seven publications), and the formula calculated M ≈ 2.62. Therefore, the authors of the 3 published papers are the core authors. The top 5 authors in terms of the number of publications in the research areas discussed in this study are listed in Table 4. The node type was selected as the author to obtain a visual knowledge map of the collaborative network of the main author, with 397 nodes, 517 connections, and a density of 0.0066 (Fig. 5). Each node represents an author and the size of the name label is positively correlated with the number of author posts. The connection between nodes represents the collaboration between authors, and the thickness of the connection indicates the strength of the collaboration. As can be seen from the map, several subsets of cooperation were formed between the main authors. The larger subsets are the 2 author groups centered on Wolf Steven L, Cramer Steven C, and Dodakian Lucy. The connection between the 2 subsets was dense, and there was significant cooperation and exchange between the authors. However, relatively little scientific research cooperation and academic exchange generally occurs among the major researchers in this field.

**Table 4:** Top 5 authors by publications.

Rank	Author	Country	Publications
1	Guidetti, Susanne	Sweden	7
2	Cramer, Steven C	USA	7
3	Lannin, Natasha A	Australia	6
4	Wolf, Steven L	USA	6
5	Ovbiagele, Bruce	USA	5

### 3.6. Keyword analysis

#### 3.6.1. Keyword co-occurrence.

Keywords include the condensation of core content in the literature. Analysis of the frequency of keywords in a research field helps researchers grasp research hotspots and directions in the research field. The node type was selected as the keyword to obtain a visual map of keyword co-occurrence. The co-occurrence analysis of key words in telerehabilitation after WoSCC from 2012 to 2022 found that, excluding search words and meanings similar to rehabilitation, stroke, recovery, telerehabilitation, stroke rehabilitation, people, and chronic stroke. The keyword with the highest frequency was virtual reality (VR) (142), followed by care (105) (Table 5). The keywords with the highest centrality were spinal cord injury (0.25), individual (0.19), efficacy (0.16), and exercise (0.15). Thus, VR technology has been a hot topic in remote rehabilitation technology research in the past 10 years. The care of remote rehabilitation participants is another popular research topic, and exercise is the main intervention method. The goal of rehabilitation interventions for stroke survivors with functional defects is to improve and cultivate patients’ self-efficacy.

**Table 5:** Top 10 keywords in frequency and centrality in co-occurrence analysis.

Rank	Keywords	Frequency	Keywords	Centrality
1	Rehabilitation	267	Spinal cord injury	0.25
2	Stroke	263	Individual	0.19
3	Recovery	156	Chronic stroke	0.19
4	Virtual reality	142	Efficacy	0.16
5	Telerehabilitation	115	Exercise	0.15
6	Care	105	Arm	0.14
7	Stroke rehabilitation	97	Technology	0.14
8	Therapy	87	Acute ischemic stroke	0.14
9	People	76	Impairment	0.13
10	Upper limb	76	Balance	0.13

#### 3.6.2. Keyword clustering.

**Figure 6.**
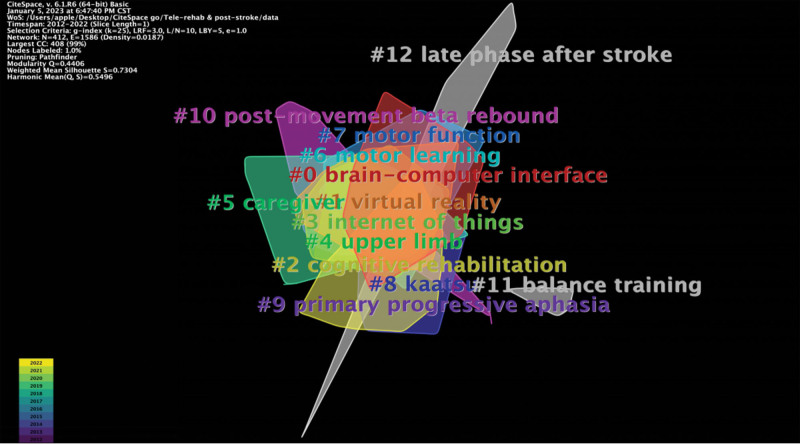
Keyword clustering mapping. # refers to the serial number of the clusters. Clustering is formed by CiteSpace by aggregating keywords as clues. The different color blocks refer to the clusters of the corresponding colors. The size of the color block is the same as the size of the cluster.

The log-likelihood rate algorithm was selected based on keyword co-occurrence, and keyword labels were used for clustering. Thirteen clusters were obtained with a cluster module value of Q = 0.4406 and a cluster average contour value (Mean Silhouette) of S = 0.5496, representing reasonable cluster results. A module value >0.3 is considered significant in the cluster structure, and the closer the S value is to 1, the higher the homogeneity of keywords within the cluster. A keyword clustering map is shown in Figure 6. The clustering results were outputted to form a keyword clustering table (Table 6). The 5 largest clusters are # 0: brain-computer interface, # 1: VR, # 2: cognitive rehabilitation, # 3: internet of T things, and # 4: upper limb. The 5 groups with the highest S values are # 12: late phase after stroke, # 11: balance training, # 9: primary progressive aphasia, # 5: caregiver, and # 10: postmovement beta rebound.

**Table 6:** Keyword cluster analysis.

Clusters	Silhouette	Size	Keywords (LLR)
#0 brain-computer interface	0.659	62	Brain-computer interface; electroencephalography; task analysis; motor imagery
#1 virtual reality	0.645	59	Virtual reality; parkinsons disease; multi sclerosis; system; improves balance
#2 cognitive rehabilitation	0.698	58	Cognitive rehabilitation; ict; covid-19; education; program
#3 internet of things	0.737	43	Internet of things; upper extremity; assistive technology; validity; physical rehabilitation
#4 upper limb	0.72	42	Upper limb; Ghana; sub-saharan Africa; locomotion; visual feedback
#5 caregiver	0.865	29	Caregiver; caregiver burden; virtual reality; older people; participation in everyday life
#6 motor learning	0.748	27	Motor learning; aging; risk; biofeedback; physical activity
#7 motor function	0.773	26	Motor function; qualitative research; occupational therapy; upper limb; telerehabilitation
#8 kaatsu	0.737	22	Kaatsu; exercise for cardiac rehabilitation; vascular occlusion training; telerehabilitation
#9 primary progressive aphasia	0.895	20	Primary progressive aphasia; patients; language; speech; telepractice
#10 post-movement base rebound	0.85	10	Post-movement beta rebound; subacute stroke; event related (de) synchronization; digital health; risk reduction
#11 balance training	0.969	5	Balance training; posture; instrument; apparatus; postural response
#12 late phase after stroke	0.974	5	Late phase after stroke; neurofilament light chain; cerebrovascular disease; stroke recovery

LLR = log-likelihood rate.

#### 3.6.3. Keyword burst.

**Figure 7.**
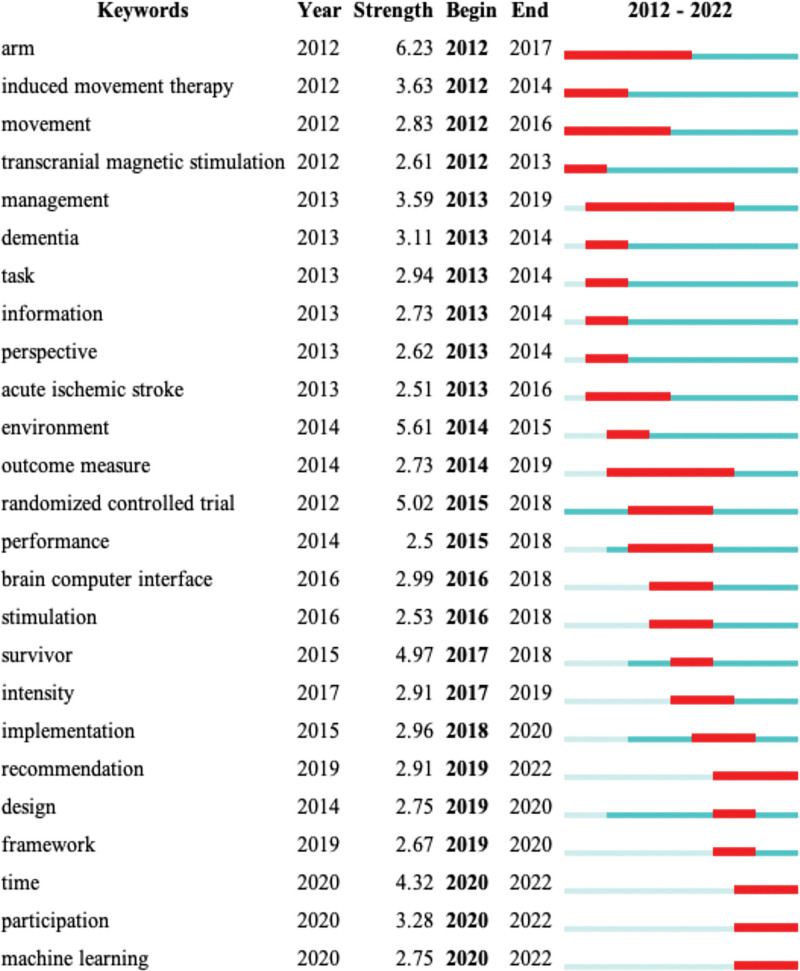
25 keywords with the strongest citation bursts. Configure the detection model: the number of states: 2; minimum duration: 2. The horizontal line represents the timeline of the year, and the red color indicates the duration of the strongest burst for the corresponding keyword, while the blue color indicates that the keyword is under research or discussion, but is less popular than the strongest burst.

Burstness keywords are those detected by CiteSpace in a certain period of time, which can, to some extent, reflect the dynamics of a certain research field and the rise or decline of a certain research direction. In the keyword co-occurrence operation panel, burstness was selected, and years per slice was set to 1, obtaining the keyword burst map of stroke telerehabilitation from 2012 to 2022 (Fig. 7). The horizontal line represents the timeline and the red line indicates the strongest period. Randomized controlled trials (RCTs), induced movement therapy, arm, movement, and transcranial magnetic stimulation have always been the subject of research, and they have reached the strongest burst intensity in different time periods. However, they have since been replaced with other keywords. Management has been a research hotspot since its emergence in 2013, and continues to be a hotspot until 2019. RCTs are a popular research method in telerehabilitation after stroke, and research focuses on upper limb exercise and induced exercise therapy. Although the burst time of the environment is short, the burst intensity is very strong, and the exercise environment may be related to the safety of patients participating in telerehabilitation intervention. The keywords that have exploded in the last 2 years are time, machine learning, and participation, and graphical analysis predicts that they will most likely be the research directions worthy of attention in the next few years.

### 3.7. Literature co-citation analysis

Two (or more than 2) articles were cited by 1 or more later papers, indicating that the 2 documents were jointly cited in the relationship. Literature with a higher co-citation frequency during a given time period is considered to have an important influence within this research field during this time period. The co-cited knowledge map can also reveal closely connected literature and common themes in similar literature. Therefore, researchers can explore the development status and change in scientific structure with the help of co-citation knowledge graphs and conduct frontier analysis and scientific research evaluation of the research field of concern. The node type reference was selected to obtain information for the literature co-citation analysis (Table 7). Tele-Rehabilitation after Stroke: An Updated Systematic Review of the Literature is the most influential publication, which was co-cited 54 times. This is followed by Guidelines for Adult Stroke Rehabilitation and Recovery: A Guideline for Health care Professionals from the American Heart Association/American Stroke Association. A guideline for health care professionals from the American Heart Association/American Stroke Association. In the top 10, there are 2 highly co-cited works by Chinese scholar Chen J.

**Table 7:** Top 10 papers with the most co-citations.

Rank	Author	Yr	Title	Frequency
1	Sarfo, FS	2018	Tele-Rehabilitation after Stroke: An Updated Systematic Review of the Literature.^[31]^	54
2	Winstein, CJ	2016	Guidelines for Adult Stroke Rehabilitation and Recovery A Guideline for Healthcare Professionals From the American Heart Association/American Stroke Association.^[9]^	40
3	Laver, KE	2020	Telerehabilitation Services for Stroke.^[32]^	37
4	Llorens, R	2015	Effectiveness, Usability, and Cost-Benefit of a Virtual Reality-Based Telerehabilitation Program for Balance Recovery.^[33]^	30
5	Chen, J	2017	Effects of Home-based Telesupervising Rehabilitation on Physical Function for Stroke Survivors with Hemiplegia A Randomized Controlled Trial.^[34]^	29
6	Cramer, SC	2019	Efficacy of Home-Based Telerehabilitation vs In-Clinic Therapy for Adults After Stroke: A Randomized Clinical Trial.^[35]^	28
7	Chen, J	2015	Telerehabilitation Approaches for Stroke Patients: Systematic Review and Meta-analysis of Randomized Controlled Trials.^[36]^	27
8	Dodakian	2017	A Home-based Telerehabilitation Program for Patients With Stroke.^[37]^	25
9	Laver, KE	2013	Telerehabilitation Services for Stroke.^[38]^	24
10	Tchero, H	2018	Telerehabilitation for Stroke Survivors: System Review and Meta-analysis.^[39]^	24

## 4. Discussion

### 4.1. Basic information

In the past 10 years, research on telerehabilitation after stroke has developed rapidly. In WoSCC, the number of publications in 2018 more than doubled compared with that in 2012, showing a yearly increase, indicating that this research field has been widely studied by researchers and has good development prospects. Australia, the United States, Canada, the United Kingdom, and China are keen on research on telerehabilitation technology combined with rehabilitation of stroke patients. Most institutions with strong academic research ability in this field are from these countries. Research on these institutions is relatively mature and has a strong academic influence. In total, Chinese scholars have published 101 articles in the past 10 years, and a number of core authors and important literature in the field has prompted China to build a foundation for stroke telerehabilitation study. Some achievements have been made, and researchers should continue to strengthen study and exchange with advanced research groups and outstanding scholars abroad to enhance academic influence and promote further development of tele-rehabilitation technology in the field of stroke.

The results of the visualization analysis indicate that research connections exist between countries, research institutions, and scholars and that some relatively concentrated collaborative networks have been formed; however, such connections are relatively limited. In general, European countries have the most intensive cooperation networks, and more frequent exchanges and cooperation. In Asia, China has published more articles, while Singapore and Pakistan are more central.

### 4.2. Study methods

According to keyword clustering and keyword burst analysis, RCTs had the strongest burst period from 2015 to 2018 and were a popular research method related to poststroke rehabilitation. In addition to RCTs, the research focus included outcome index measurements and trial design. Chen J single-blind RCT randomly assigned stroke survivors to a home remote-monitoring rehabilitation group or a routine rehabilitation group. The participants were evaluated at baseline, postintervention week 12, and follow-up week 24 using the Barthel index, Berg balance scale, modified Rankin scale, and caregiver strain index. The results showed that home-based remote monitoring rehabilitation is likely as effective as routine outpatient rehabilitation in improving the functional recovery of stroke survivors and can reduce the burden of caregivers.^[34]^ A randomized longitudinal controlled trial, designed by Spanish, Swedish and French hospitals, evaluated whether a digital health-based rehabilitation game system intervention combined with traditional care is superior to traditional care alone for stroke patients in hospital and home recovery.^[40]^ This study is scheduled to be completed in July 2023, and the trial authors predicted improved recovery, higher acceptance and lower costs due to a soft landing from clinic to home rehabilitation. In addition, the trial results may identify new criteria for online and remote assessment, diagnosis, and intervention in European hospitals. Although many studies have tested the efficacy of telerehabilitation through RCTs, conclusions about the effect of this strategy are not easily drawn because interventions and compared subjects vary widely in these studies.^[41,42]^ Blinding participants is difficult in these studies, which will affect the persuasiveness of the trial conclusions to some extent.^[34,43]^ In randomized controlled studies in this field, researchers need to ensure quality control as much as possible, including inclusion and exclusion criteria, scientific randomization, control of bias, and scientific data statistics.^[44,45]^ Palmcrantz et al^[46]^ introduced an interactive remote rehabilitation technology through a mixed research method and explored its advantages and disadvantages, proved the feasibility and safety of the technology, and provided guidance for further development and testing of interactive remote technology for home rehabilitation. RCTs can be predicted to remain an important research method in the field of stroke rehabilitation, and an increasing number of researchers will select mixed research methods in the future because of their advantages.

### 4.3. Research hotspots and frontiers

Remote rehabilitation has advanced with the development of telemedicine. Mobile health and digital health technology are both emerging telemedicine service modes that combine rehabilitation medicine technology with computer technology, communication technology and mobile network technology.^[40,47]^ The study of telerehabilitation has been closely integrated with electronic science, telecommunications and sports science. Therefore, research on VR technology and rehabilitation robot technology has become a hot topic in remote rehabilitation after stroke. Brain-computer interface technology, which detects motion images from the electroencephalogram, has been further developed in a project from Singapore.^[48]^ Chen J et al^[34]^ provided real-time video conferencing, education, consultation, neuromuscular electrical stimulation, electromyography parameter collection, and other remote rehabilitation services for stroke patients through a remote rehabilitation system integrated with electromyography-triggered neuromuscular stimulation. Maddahi et al^[49]^ introduced a portable remote rehabilitation mobile platform that includes a robotic health glove developed by the researchers, a cell phone application that employs wireless network technology, video transmission capabilities, supplemented by task-oriented exercise tasks. Spits et al^[50]^ linked a robotic assistive device (Arm Assist) to an Antari home care platform to help patients train in upper extremity function in the home environment. The therapist achieves the desired results with the help of the biomechanical data collected by the system and with remote communication and remote monitoring of the patient on the platform.^[50]^ Mura et al^[40]^ developed a cloud-based rehabilitation game system that combines VR, motion capture, and wearable devices to enable artificial intelligent rehabilitation. The system provides rehabilitation exercises in specific, goal-oriented, and task-specific forms of action.^[40]^ VR technology can provide individualized treatment for stroke survivors with highly repetitive task-specific movements, specific feedback, and carried out in a rich environment, which has improved patient satisfaction with rehabilitation to some extent. However, the current VR technology still needs to be improved, such as increasing the variety of games, enriching the picture level, enhancing the realism of training, and addressing the problem of monotony. Similar to VR technology, rehabilitation robots offer advantages over traditional rehabilitationco-. However, even multi-degree-of-freedom robots cannot currently move all joints. It is predictable that VR and rehabilitation robots will be an important part of the future of intelligent rehabilitation.

The remote supervision, professional rehabilitation guidance and task training interest provided by the remote rehabilitation project can all help to cultivate the self-efficacy of patients after stroke, improve patients’ self-health management willingness, and increase the enthusiasm of patients to participate in rehabilitation exercise. Owing to bad mood, fatigue, pain, lack of health-related knowledge, and lack of supervision and motivation factors, stroke survivors have poor functional exercise compliance,^[51]^ and researchers have noticed this characteristic. Future studies should implement measures to improve rehabilitation participation and exercise enthusiasm in remote intervention programs.

When telerehabilitation is conducted, therapists and patients provide guidance and advice to patients through online interactions, while caregivers indirectly participate in or directly assist patients in rehabilitation activities. The importance of caregivers in telerehabilitation interventions for stroke survivors has been noticed by investigators.^[52]^ Previous studies have shown that caregivers of stroke patients are generally anxious and depressed, and they should be given psychological counseling and more social support.^[53,54]^ Due to the increasing focus on adverse events in telerehabilitation interventions, caregiver care capacity, patient safety risk identification, and the role of caregivers in telerehabilitation interventions should continue to be of interest in future studies.

Studies have suggested that participants’ exercise adherence is lower if the prescribed sessions are too frequent (6–7 days/week) or last too long (12 weeks).^[55]^ The time and intensity of participation in telerehabilitation exercise after stroke is also related to patient safety and the effectiveness of rehabilitation intervention. Therefore, the time and intensity of exercise in remote interventions should follow the relevant guidelines and recommendations, but researchers need more high-quality, in-depth research. In short, VR and rehabilitation robots will be a valuable research direction in poststroke telerehabilitation. In addition to facing hardware, software, and ergonomic barriers, telerehabilitation techniques should improve the efficacy and safety of the intervention. Furthermore, cost-effectiveness should be included in the evaluation scope, which is the key to the feasibility of telerehabilitation techniques. Owing to the characteristics of remote rehabilitation and technology-driven development, developed countries, especially those that already have a good research foundation, are believed to be more likely to take the lead in making influential breakthroughs in research in this field.

### 4.4. Outlook

A review of the literature shows that most research subjects of telerehabilitation after stroke had strict restrictions, and patients with more than a moderate or severe stroke degree were excluded. At present, a few explored telerehabilitation techniques are designed for patients with moderate and severe stroke, and research in this direction may also be a major difficulty in the study of telerehabilitation after stroke. In addition, although some studies investigated participants’ satisfaction with the telerehabilitation program, system and safety after the intervention, the evaluation methods and tools varied, and the development of evaluation tools for the telerehabilitation intervention model is warranted.^[46,56]^ Finally, the practice patterns of tele-rehabilitation services after stroke are diverse.^[57–60]^ In this case, unified guidelines or standards are needed to ensure and promote high-quality healthcare services. A scoping review found that most guidelines for tele-rehabilitation programs do not specifically address movement-related assessments and cannot effectively address the limitations of remote physical assessments, especially when patients are unable to move their bodies or have limited mobility.^[61]^ Remote physical examination is another major challenge for remote assessment. Guidelines and implementation processes regarding the safety and accuracy of remote assessments and remote physical examinations are necessary, and while some research in this area has been conclusive, it is still insufficient.^[62,63]^

## 5. Conclusions

In the past 10 years, research on telerehabilitation technology in the field of stroke survivors rehabilitation has developed rapidly and gained a relatively mature research basis, but higher quality research and more reliable evidence to show that the efficacy of telerehabilitation is better than or equivalent to that of traditional rehabilitation methods is still necessary. Telemedicine services for stroke rehabilitation are interdisciplinary and involve professional research based on computer technology, mobile communication technology, and electronic information technology. VR technology and rehabilitation robotics are already destined as popular directions for the future development of tele-rehabilitation. Moreover, the choice of exercise duration and intensity needs to be supported by more valuable scientific evidence from future studies. The safety and cost-effectiveness of poststroke telerehabilitation intervention are also issues that cannot be ignored by researchers, which ensures the generalizability of research results. Previous studies will help to explore the rehabilitation modalities and efficacy of tele-rehabilitation technologies for patients with moderate-to-severe stroke. Researchers worldwide should combine their own characteristics and advantages, strengthen academic exchanges and cooperation with advanced foreign research institutions or research teams, achieve scientific breakthroughs, and explore poststroke remote rehabilitation technologies and service models suitable for promotion in different environments to improve the quality of survival and living standards of stroke survivors.

## 6. Limitations

Only the WoSCC database was included as a source of record collection; consequently, the present analysis does not reflect the full context of the research area explored. CiteSpace, developed by Professor Chen Chaomei at Drexel University, was used as the analytical tool in this study. Because of the software itself, some subtle data may be biased, and the interpretation criteria of the visual map may not be completely consistent, which will also have a certain impact on the data analysis results. We aim to improve the accuracy of the data and interpret them in subsequent studies.

## Acknowledgments

We would like to thank the editor and anonymous reviewers for their useful comments on earlier drafts.

## Author contributions

**Conceptualization:** Yan Sun.

**Data curation:** Linzhang Li.

**Formal analysis:** Linzhang Li, Yan Sun.

**Funding acquisition:** Yan Sun.

**Methodology:** Yan Sun.

**Supervision:** Yan Sun.

**Validation:** Linzhang Li.

**Visualization:** Linzhang Li.

**Writing – original draft:** Yan Sun.

**Writing – review & editing:** Linzhang Li, Yan Sun.
